# Existing Policies/Guidelines on the Environmental Dimension of Antimicrobial Resistance in India: An Insight into the Key Facets through Review and SWOT Analysis

**DOI:** 10.3390/tropicalmed7110336

**Published:** 2022-10-29

**Authors:** Falguni Debnath, Debjit Chakraborty, Sandip Giri, Shatabdi Saha, Soume Pyne, Raja Chakraverty, Agniva Majumdar, Alok Kumar Deb, Vishal Diwan, Rajesh Bhatia, Shanta Dutta

**Affiliations:** 1 Indian Council of Medical Research (ICMR), National Institute of Cholera & Enteric Diseases, Kolkata 700010, India; 2 Indian Council of Medical Research (ICMR), National Institute for Research in Environmental Health, Bhopal 462030, India; 3 Antimicrobial Resistance (AMR) Expert, Food & Agriculture Organization, New Delhi 110003, India

**Keywords:** antimicrobial resistance, environment, policy, guidelines, legislations, SWOT analysis

## Abstract

Background: Antimicrobial resistance (AMR) is a multidimensional phenomenon. The environment acts as a mixing pot of drug-resistant bacteria from many sources such as pharmaceutical, biomedical, veterinary, and agricultural sectors. In this study, we analysed the existing AMR-related policies/guidelines/legislations in India in the above domains and how the current practices are being guided by them. Methods: We used a convergent parallel mix method design. Quantitative data were collected through a review of policies/guidelines/legislations in the said domains and analysed using the SWOT tool parallelly supported by key informant interviews of domain-specific stakeholders. Results: Altogether, 19 existing AMR policies/guidelines/legislations were identified. The existence of few policies/guidelines in each domain indicated the evolving environment for policy interventions. However, the lack of capacity among farmers, inadequate provision for structured capacity building, high cost of alternatives to antimicrobials, and lack of provision of incentivisation in case of crop failure were identified as the major weaknesses prevalent across the domains. Opportunities for policy refinements/the introduction of new policies are ample. However, easy access to antimicrobials and injudicious use imposes threats to AMR containment in all sectors. Conclusions: Despite having a few policies for the containment of AMR, their implementation witnesses challenge due to the lack of collaborative approaches, the existence of policies disjointed from ground reality, infrastructural issues, and the lack of capacity and resources.

## 1. Introduction

Since the introduction of antibiotics in 1930, they have gained immense importance in global health and food production [1]. However, the irrational use of antibiotics fosters the process of the mutation, selection, and proliferation of bacteria in the environment, resulting in antimicrobial resistance (AMR), which is one of the major public health concerns of recent times [2]. The indiscriminate and extensive use of antimicrobials is known to be the biggest driver of AMR, and according to Klein et al., low- and middle-income countries (LMICs) tend to consume antibiotics more than high-income countries [3]. 

AMR is a multifaceted and complex global phenomenon, and the environment has been considered a mixing pot of drug-resistance bacteria from all sources [4]. The pharmaceutical, biomedical, veterinary, and agricultural sectors are considered potential sources of AMR pollution in the environment [5,6,7,8,9]. The application of antibiotics is not only restricted to therapeutics but is also used as prophylactics [10]. To meet the mounting demand for food for the growing population, the rampant use of antibiotics as prophylactics and growth promoters in animal husbandry, aquaculture, and agriculture is increasing in low- and middle-income countries, including India [11]. Unfortunately, India recorded the highest consumption of antibiotics, indicated by their increasing sales [12]. Prompt intervention is needed to address the AMR issue. Moreover, inadequate sanitation and poor hygiene practices amplify the propensity for increasing antimicrobial pollution in the environment [13]. AMR is being recognised as one of the barriers to the attainment of Sustainable Development Goals (SDG 3: good health and well-being and SDG 6: clean water and sanitation). 

In recent times, AMR has been recognised as a “One Health” problem due to its interconnectedness between human and animal health and the environment. The burden of antimicrobial-resistant bacteria also varies with geographical location, further catalysed by different environmental variables [14] and seasonality [15]. Moreover, there are several social determinants such as poverty, illiteracy, and rapid urbanisation that perpetuate the spread of contagious bacterial diseases, resulting in increased susceptibility to infectious diseases in the Indian Subcontinent [16]. This interconnectedness of humans, animals, and the environment brings about the complexity of AMR, which needs to be addressed through multicentric, collaborative approaches. 

As a global initiative to contain AMR, the WHO, along with the FAO and the OIE, developed a comprehensive Global Action Plan (GAP) on AMR in 2015 [17]. Additionally, in 2019, the WHO categorised antibiotics in the AWaRe groups to monitor their administration. However, its implementation across LMICs is still awaited [18]. Inadequate empirical evidence on AMR incidences in the different sectors and their interconnectedness poses a major hindrance to unravelling the environmental dimension of AMR and demands a review of the existing policies and guidelines, and the formulation of new ones if required. Against this backdrop, we conducted situation/SWOT analyses on the existing AMR-related policies/guidelines/legislations in India and how they are guiding the current practices.

## 2. Methodology of the Study

### 2.1. Study Design

We used a convergent parallel mix method design in the present study, where the quantitative data were collected through a review of policy-related documents/guidelines/legislations in various priority domains mentioned earlier, all of which are related to the environmental dimensions of AMR. The qualitative data were concurrently collected using key informant interviews of relevant stakeholders identified from similar priority domains and available during the period of study.

### 2.2. Study Duration

The study was conducted during the period of April 2021–March 2022. 

### 2.3. Study Population

We considered all the legislations, policies, and guidelines related to the use of antibiotics/antimicrobials; the allowable limits of antibiotics/antimicrobials in various items; the discharge limits of antibiotics from factories; waste management; and the monitoring of antimicrobial use in plant agriculture/animal husbandry/human health sector. Key informant interviews (KIIs) were conducted with stakeholders of the aforesaid priority domains at different levels, i.e., farmers, practitioners, executive or implementing officers/scientists, and policymakers. 

### 2.4. Literature Review

We initially searched the official websites of various line departments of the Government of India for the policies/guidelines/legislations directly or indirectly related to the containment of antimicrobial resistance in India. The electronic databases of the published literature such as PubMed/Medline, Google Scholar, Scopus, Embase, and Index Medicus were also searched for this purpose for the last ten years (2010–2020) (Table S1). Simultaneously, we also received information about such existing policies during the KIIs and incorporated it into our studies. 

### 2.5. Interview with Stakeholders

The KIIs were conducted to understand how the current AMR policy landscape in India is guiding the practices and what could be the possible way forward. The key informants were selected based on their expertise and willingness to share information. A total of 17 KIIs were conducted in this study. The process of KIIs was initiated with the development of an interview guide for each domain encompassing key questions in the following segments: the existing policies/guidelines/legislation in India related to AMR containment in various domains; the status of implementation of those policies/guidelines/legislations; the challenges in implementing them; the gaps in the existing intervention strategies; and the way forward to improve the environment to contain AMR in India. The interviews were audio-recorded. Data were transcribed into text data, then arranged systematically, and coded based on the predetermined scopes (the current landscape of policies/guidelines; implementation challenges; gaps if any; current practices; the area of intervention) identified through the literature review. The defined codes were then gathered together by eliminating redundancy to develop the key themes. Until this point, two investigators ran this process independently. 

### 2.6. SWOT Analysis

The SWOT (strength, weakness, opportunity, and threat) analysis is one of the tools that can be used for strategic management to appraise policies. We performed a SWOT analysis in this study to strategically identify the scopes of intervention in the mentioned domains for containing AMR in the environment in India, as this analysis considers both the internal and external factors and generates information for prioritising and facilitating decision making. 

## 3. Results

In this study, we found 19 existing AMR policies/guidelines/legislations in India. Two were from the plant/agricultural sector, eight from animal husbandry, five from the human health sector, and four from the pharmaceutical sector. 

### 3.1. Policies/Guidelines/Legislation Reviewed in the Plant/Agricultural Domain

Two existing policies related to AMR were found in this domain. One was an act related to the use of insecticide along with antibiotics, which came into force in 1968 [19]. The act mandated the licensing of the production and trading of antibiotics. Another one was implemented by the Ministry of Agriculture and Farmers Welfare, Government of India, in 2021 [20], regarding the use of prescribed formulations of insecticides, fungicides, and antibiotics (Table 1).

#### Analysis of the Reviewed Policies in the Plant/Agricultural Domain (Table S2)

**Figure 1:**
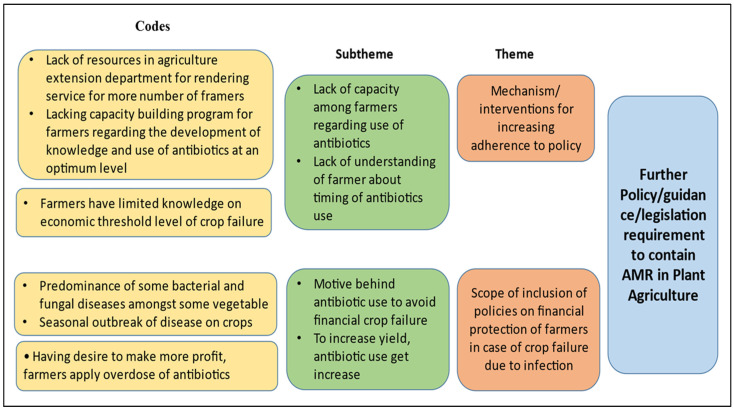
Thematic areas for intervention in plant/agricultural sector.

We conducted a SWOT analysis of the policies available in this sector. It is evident from the presence of current policies that the system has started to evolve. However, implementation challenges persist. The ignorance among farmers regarding the optimum use of antimicrobials and the desire to make a profit are major weaknesses. During the KIIs, one of the key informants (district-level agricultural officer, male) mentioned, “the farmer believes that application of antibiotic in large amount keep the crops healthy and increase their yield, though the disease occurred in crops can be manageable also without any economic loss”. Another key informant (scientist, male) mentioned, “the farmers cannot identify the acute stage of infection when the antibiotic formulation is needed to apply on crops”. However, these weaknesses can also be used as opportunities for new policy introduction focusing on the capacity building of farmers. However, the absence of surveillance on sales of such preparation at the market may currently act as a threat to optimising the use of antimicrobials in the plant/agricultural sector (Figure 1).

### 3.2. Policies/Guidelines/Legislations Reviewed in the Veterinary Domain 

We reviewed 14 policies/ guidelines/ legislations related to AMR in the veterinary sector (Table 2). Out of them, the majority are implemented by the Ministry of Health and Family Welfare, Government of India, and a few by the Ministry of Commerce and Industry, GOI, and the Department of Animal Husbandry and Dairying. Many of them are related to limiting the use of antibiotics for consumable animal products and processed animal food, and few are related to stringent surveillance mechanisms to regulate the use of antibiotics, monitor antibiotic residue levels in milk and honey, and monitor the presence of antibiotic residues in exportable aquaculture products [21]. The National Animal Diseases Control Programme (NADCP) was implemented in 2019 by the Department of Animal Husbandry and Dairying (DAHD) for controlling foot and mouth disease and brucellosis among farm animals (cattle, buffalo, sheep, goat, and pig) through vaccination [22]. The Indian Network of Fisheries and Animal Antimicrobial Resistance (INFAAR), founded in 2018, was designed for identifying AMR in various animal food production systems [22]. Apart from that, a surveillance mechanism known as the National Animal Disease Reporting System (NADRS) was developed by the DAHD [22]. 

#### Analysis of the Reviewed Policies in the Veterinary Domain (Table S2)

**Figure 2:**
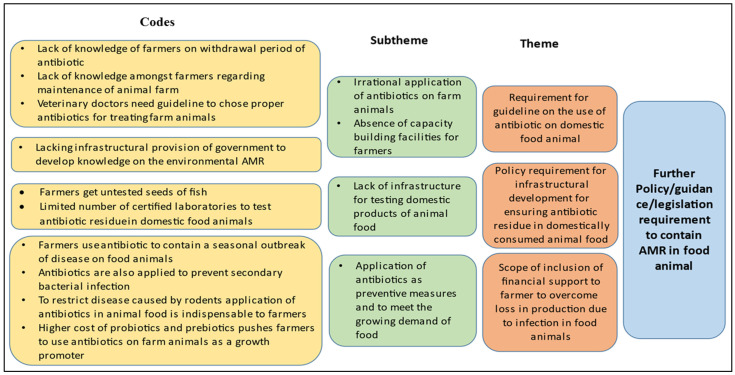
Thematic areas for intervention identified through KIIs in animal health sector for containment of AMR.

Upon analysis, it is understood that the concerned ministries are aware of the problem and already have started acting on it. However, the lack of provisions for quality checks of domestic products even seeds, the absence of structured capacity-building programmes for farmers, the absence of surveillance mechanisms for regulation of the administration of antibiotics and mechanisms for the regular checking of antibiotic residues in domestic food animals and monitoring of critically important antibiotics for humans which are used in animals as growth promoters are identified to be major weaknesses. Moreover, the lack of coordination among different authorised bodies, such as the Bureau of Indian Standards (BIS) and the Food Standards and Safety Authority of India (FSSAI) regarding the list of antibiotics to be used within a limit, may mislead the stakeholders on the administration of antibiotics on farm animals. One of the key informants (representative of farmers, male) from this sector mentioned, “The farmers don’t get good quality seeds from agents and even farmers are completely unaware about the SPF (Specific Pathogen Free) testing reports of the seeds. Farmers do not have enough knowledge on farm management and treatment of animals, so they use antibiotics as preventive measures and apply those as they want”. However, the presence of strict surveillance on the use of antimicrobials for exportable food animals is the opportunity to introduce similar mechanisms for quality checks in domestic food animals. Presently, the absence of any provision for controlling the high cost of available growth promoters of animals acts as a threat, as it leaves no choice for the farmers but to use antibiotics as growth promoters (Figure 2). Another key informant (representative of farmers, male) from this sector mentioned, “As probiotics and prebiotics are very expensive to our farmers they tend to use antibiotics as a growth promoter of animals”. Hence, despite the presence of so many policies in this field related to the containment of AMR, animal food remains a major source of transmission of AMR bacteria in the environment.

### 3.3. Policies/Guidelines Reviewed and Analysed in Human Health Sector

The Ministry of Health and Family Welfare, Government of India, along with its line departments such as the Indian Council of Medical Research (ICMR) and the National Centre for Disease Control (NCDC), have adopted some approaches regarding the rational use of antimicrobials in humans to diminish the emergence of multidrug-resistant bacteria and their propensity of transmission in humans (Table 3).

The presence of a national action plan for AMR (2017–2021), few guidelines for infection control, and judicious antimicrobial use sets the field to develop policies to streamline the problem of AMR in the human health sector which will eventually reflect in other sectors too. However, the lack of policies mandating the rational use of antibiotics, weak regulatory control on the over-the-counter (OTC) sale of antibiotics, and the absence of structured utilisation of available platforms for generating public awareness on the consumption behaviour of antimicrobials are major weaknesses. The pressing demand for using information technology-based monitoring and surveillance on the administration of antibiotics in humans and AMR incidents in hospitals can be used as opportunities to create national-level data on it. Moreover, addressing the determinants of AMR in a national programme mode may create an environment in the country where all the states give due importance to the problem of AMR and bring out meaningful public health action. However, its absence in the country is currently acting as a major threat. 

### 3.4. Policies/Guidelines Reviewed for the Biomedical and Pharmaceutical Sector

We reviewed four policies/guidelines/legislations related to the pharmaceutical and biomedical waste management sector (Table 4). However, the current guidelines/policies/legislations are mainly related to the disposal and management of hazardous substances and other solid and liquid wastes. Only one guideline mentions the permissible level of antibiotic residues [36].

#### Analysis of the Reviewed Policies in the Biomedical and Pharmaceutical Sector (Table S2)

**Figure 3:**
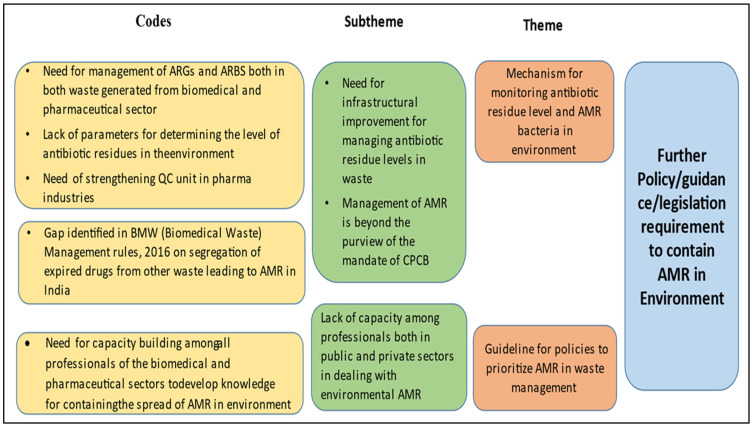
Thematic areas for intervention in biomedical waste management and pharmaceutical waste management.

Upon analysis, it is evident that newer policies can be introduced in both of these sectors, as few policies already exist, and there is a felt need for more. However, it is observed that the policies are mostly related to the disposal and management of hazardous substances and other solid and liquid wastes. This is a weak area where more clarity is required on the process of the disposal and management of antimicrobial drugs and related waste generated from hospitals, communities, and manufacturing units. In this regard, a hospital management personnel said, “All the discarded drugs are kept in the same place and sent to Central store and then outsourced further by a third party agency”. The Ministry of Environment, Forest, and Climate Change (MoEFCC) has very recently published the permissible level of antibiotic residues in effluent [36], and this may work as an opportunity to control the spread of AMR through the discharge of effluents from pharmaceutical companies. Moreover, some pharmaceutical companies have also targeted zero liquid discharge. A key informant (scientist of a pharma company, male) mentioned, “Presently our company and all other manufacturing units aim at zero liquid discharge to recycle effluents discharged in pharmaceutical manufacturing units”. However, the lack of capacity among professionals across sectors to recognise the containment of AMR as a multisector initiative poses a major threat in the current scenario (Figure 3).

## 4. Discussion

The findings of the present study depicted the current status of AMR-related policies, guidelines, and regulations in India across different sectors and the major strengths, weaknesses, and threats present in the context of the available ones. However, the study also identified domain-specific opportunities for new policy frameworks or refinement of the existing ones. 

A mechanism for the continuous capacity building of farmers remains a major requirement in all sectors. For example, it has been revealed by the Centre for Science and Environment (CSE) that the farmers in Delhi and Punjab rampantly use streptocycline as a fungicide for crop production [40]. In a few sectors, not only the lack of knowledge but also the threat of impending economic loss creates an environment for excess antimicrobial use. Despite having guidelines in a few sectors, the overuse of antibiotic formulations cannot be checked due to their easy availability in the market and the prevailing misperception of antibiotics being growth promoters. However, with the worldwide advancement of technologies for disease control in agriculture, a trend has recently been commenced in India to facilitate farmers with disease-resistant cultivators [41], but it needs to be utilised extensively to reduce the use of antibiotics. To maintain the sustainability of agriculture and food quality, the application of *Trichoderma* sp. as biopesticide and plant growth promoter agents [42] and *Pseudomonas fluorescens* [43] as biocontrol in place of antibiotic formulations can be accepted as cost-effective methods. Specifically, in the context of plant/agriculture, the adoption of biosecurity approaches such as purchasing plant materials from reputed sources, certified fertilisers, the use of disinfectant equipment, and the regular monitoring of crops [44] may present alternative strategies for better crop production. 

Research and development are required in all sectors regarding the social implication of antibiotic use and to design interventions for behavioural change among stakeholders [45]. However, to encourage small-scale and marginalised farmers to comply with rules and guidelines, provisions for subsidies or incentives for them may be created within the scope of the existing policies or new policies for each sector. 

Policies regarding investment in the research and development of cost-effective newer methods of cultivation are required to provide some alternatives to the current practice of antimicrobials as therapeutics or growth promoters, and this need has been felt across sectors. 

In the veterinary sector, through the reinforcement of the existing policies, domestically consumed products must also be regulated for antibiotic residues, as it is carried out for exportable products, and the FSSAI may be identified as the agency to bring this change. However, regarding biomedical waste management, it is time to mandate the establishment of effluent treatment plants (ETPs) at hospitals and assess them to generate data on antimicrobial residues in effluents. Guidelines on maintaining a database on the usage of antibiotics in hospitals and other healthcare units, along with AMR cases, have become important for promoting the judicious use of antibiotics. The 2016 and 2018 Biomedical Waste Management (BWM) Rules did not incorporate the segregation of antibiotics from other solid waste. Therefore, antibiotic segregation from other solid wastes should also be given priority in the revised guidelines. 

In the pharmaceutical industry, policies are required for measuring the magnitude of AMR bacteria before the effluents undergo the recycling process. Continuous monitoring for priority pathogens in environmental samples can guide policymakers regarding policy refinements on antimicrobials. 

This study suffers the limitation of evaluating the gamut of policies/guidelines present in the important domains that contribute to environmental AMR in India which may be further investigated in each domain. However, this overall analysis generates evidence on how the existing policies guide the current practices and prepares the field for further discussion on policy requirements for the containment of environmental AMR in India. 

We conclude that though public health is the prime development indicator of our country, and the government allocates abundant funds for the accomplishment of national health policies, their implementation witnesses challenges due to the lack of collaborative approaches, the existence of policies disjointed from ground reality, infrastructural issues, and the lack of capacity and resources. 

This study highlighted the requirement of effective policies and implementable guidelines as well as coordination between state and central government agencies to address the present knowledge gaps on AMR to boost India’s potential towards leadership for limiting AMR across sectors. 

## Figures and Tables

**Table 1:** Existing policies/guidelines/legislations for restricting antibiotic usage on plant agriculture in India.

Rules and Regulations/Guidelines	Implementing Agency	Highlights	Sources
The Insecticides Act, 1968		Prohibit the importing, manufacturing, and selling of insecticides and their incorporation into some antibiotics such as streptomycin and tetracycline, by authorising the registration of insecticides and licensing of the respective persons Cancellation of licenses subject to infringement.	Insecticides Act, 1968 (https://legislative.gov.in/sites/default/files/A1968–46.pdf) [19]
Central Insecticide Board and Registration Committee, Department of Agriculture, Cooperation and Farmers Welfare, Ministry of Agriculture and Farmers Welfare	Ministry of Agriculture and Farmers Welfare	Prescribed the formulation of fungicides and their combined use, including antibiotic formulation, for specific plants.	Central Insecticide Board and Registration Committee, 2021 (http://ppqs.gov.in/sites/default/files/2._mup_fungicide_upto_30.06.2021.pdf) [20]

**Table 2:** Rules, regulations, policies, and guidelines to contain AMR in aquaculture and livestock products in India.

Rules and Regulations/Policies/Guidelines	Implementing Agency	Highlights	Source
Amendments to the Drug and Cosmetics Rules in 2006, 2010, 2013, and 2015 regarding consumable animal products	Ministry of Health and Family Welfare, Department of Health, Government of India	Labelling of withdrawal periods Prohibition of the usage of antibiotics (Schedule H and H1 drugs) in food animals without a prescription Inhibition of promotional advertisement of Schedule H, H1, and X antibiotics if not endorsed by the government	Centre for Disease Dynamics, Economics and Policy, 2016 (https://cddep.org/wp-content/uploads/2017/06/india_abx_report-2.pdf) [21]
Amendments of the Drug and Cosmetics Rules in 2002 and 2003 regarding processed animal food		Delimit the use of certain antibiotics and the level of antibiotic residue in frozen processed foods	
Coastal Aquaculture Authority (CAA) Act, 2005		Banned the application of 20 antibiotics and pharmacologically active substances * in shrimp cultivation Larval and farm feed, feed additives, chemical substances and drugs, probiotics, fertilisers, immune stimulants, etc. could only be enlisted if verified as antibiotics free	Guidelines annexed to CAA rules, 2005 (http://caa.gov.in/uploaded/doc/Guidelines-Englishnew.pdf) [23]
Residue Monitoring Plan by the Export Inspection Council (EIC), 2011 and 2019	Ministry of Commerce and Industry	Surveilling the residues of antibiotics in animal food, especially milk and honey ** Non-compliance by the exporter is subjected to corrective action	Export Inspection Council, 2011 and 2019 [24,25]
Marine Products Export Development Authority (MPEDA)	Ministry of Commerce and Industry	ELISA screening laboratories for testing the presence of banned antibiotic residues such as chloramphenicol and nitrofuran metabolites in aquaculture pre-harvest produce (shrimp) National Residue Control Plan (NRCP) is a statutory requirement to export to EU countries for which samples are collected from hatcheries, feed mills, aquaculture farms, and processing plants of maritime states and tested for the presence of any antibiotic residue/contaminant	MPEDA, (https://mpeda.gov.in/?page_id=1088) [26]
National Policy for Containment of Antimicrobial Resistance, 2011	Ministry of Health and Family Welfare	Development of suitable protocols for antibiotic use in food animals Inhibiting the usage of non-therapeutic antibiotics in animals Regulation of the sale of antibiotics, especially Schedule H1 antibiotics without the prescription of certified practitioners	Srivastava, 2011 (https://main.mohfw.gov.in/sites/default/files/3203490350abpolicy%20%281%29.pdf) [27]
Chennai Declaration (2012)—A Roadmap to Tackle the Challenge of Antimicrobial Resistance	Clinical Infectious Diseases Society	Withdrawal period to minimise the existence of antibiotic residues in milk and meat Monitor the usage of antibiotics and their residues in animal food Banned OTC (over-the-counter) sale of antibiotics	Centre for Disease Dynamics, Economics and Policy, 2016 (https://cddep.org/wp-content/uploads/2017/06/india_abx_report-2.pdf) [21]
Agricultural Marketing Information Network (AGMARK)	The Directorate of Marketing and Inspection, Ministry of Agriculture and Farmer Welfare	Measured the level of antibiotic residues in frozen raw meat and eggs of the assigned producers	
National Animal Disease Control Programme (NADCP)	Department of Animal Husbandry and Dairying	Controlling Foot and Mouth Disease and Brucellosis amongst farm animals (cattle, buffalo, sheep, goat, and pig) through vaccination	Kumar et al., 2021 (https://journals.lww.com/ijmr/Fulltext/2021/03000/Animal_disease_surveillance__Its_importance__.10.aspx) [22]
Indian Network of Fisheries and Animal Antimicrobial Resistance (INFAAR), 2018	Indian Council of Agricultural Research in collaboration with the Food and Agriculture Indian Council of Agriculture Research Organization (FAO) and the United States Agency for International Development (USAID)	Detecting the AMR in various animal food production systems Describes the spread of resistance and hypothesises the origin of resistant bacteria by means of a national surveillance program	
National Animal Disease Reporting System (NADRS)	Department of Animal Husbandry and Dairying	Records and monitors livestock disease situation in India to undertake preventive and curative action swiftly during disease emergencies	
National Animal Disease Referral Expert System (NADRES)	ICAR—National Institute of Veterinary Epidemiology and Disease Informatics	Gathering information on animal diseases including associated risk factors in a particular duration of time	
National Centre for Disease Control		Cessation of widespread consumption of antibiotics in animals for non-therapeutic processes	Centre for Disease Dynamics, Economics and Policy, 2016 (https://cddep.org/wp-content/uploads/2017/06/india_abx_report-2.pdf) [21]
Central Drugs Standard Control Organization			
Directorate General of Health Services			
Bureau of Indian Standards		Discontinuation of antibiotic application in poultry feed as a growth promoter due to having systemic action on intestines in five years Recommended not to use chloramphenicol, doxycycline, tetracycline, nitrofurans, furazolidone, etc., as growth promoters.	
Department of Animal Husbandry, Dairying, and Fisheries, the Ministry of Agriculture		Suggestions for complying with the prescription of veterinarians regarding the usage of antibiotics Orientation of different departmental personnel and the stakeholders on the prudent use of antibiotics	
Food Safety and Standards Authority of India		Published revised list of antibiotics with restricted usage on food animal Chloramphenicol, doxycycline, nitrofurans, and furazolidone are not listed for veterinary use	
Central Pollution Control Board		Proposed disposal mechanism of manure and litter of dairy and poultry farms to minimise the effect on the environment	CPCB (Dairy), 2021 (http://www.uppcb.com/pdf/Reviesd-Guidelines_290721.pdf) [28] CPCB (Poultry), 2021 (https://cpcb.nic.in/openpdffile.php?id=TGF0ZXN0RmlsZS8zMzFfMTYyOTA5OTQwMF9tZWRpYXBob3RvMjg2MjcucGRm) [29]
National surveillance Programme for Aquatic Animal Diseases (NSPAAD), 2015	ICAR-Central Brackish Water Aquaculture	Monitoring infectious diseases of aquatic animals in brackish water aquaculture	Vijayan, 2016 (http://www.ciba.res.in/Books/cibaspl83.pdf) [30]
Consortia Research Project on Development of Vaccines and Diagnostics (CRP on V&D), 2015	ICAR-Central Brackish Water Aquaculture	Cost-effective and sensitive tools for disease diagnosis and prophylactic measures are being developed	

* **CAA enlisted banned antibiotics and pharmacologically active substances**: chloramphenicol, nitrofurans including furaltadone, furazolidone, furylfuramide, nifuratel, nifuroxime, nifurprazine, nitrofurantoin, nitrofurazone, neomycin, nalidixic acid, sul-phamethoxazole, Aristolochia spp and preparations thereof, Chloroform, chlorpromazine, colchicine, dapsone, dimetridazole, metronidazole, ronidazole, ipronidazole, other nitroimidazoles, clenbuterol, diethylstilbestrol (DES), sulfonamide drugs (except approved sulphadimethoxine, sulphabromomethazine, and sulphaethoxypyridazine), fluoroquinolones, and glycopeptides. ** **Export Inspection Council (EIC) delimited residue of antibiotics for milk products**: sulphonamides, tetracyclines, nitrofu-rantoin, aminoglycosides, anthelminthic, nitroimidazole, anthelmintic, beta-lactam, doxycycline, enrofloxacin, chloramphenicol, erythromycin, spiramycin, thiamphenicol, tilmicosin, trimethoprim, and tylosin. **For honey**: sulphonamides, tetracyclines, beta-lactam, macrolides, imidazole, dihydrostreptomycin, and ciprofloxacin.

**Table 3:** Guidelines on the rational use of antibiotics on human beings.

Guidelines	Implementing Agency	Highlights	Source
National Programme on AMR Containment (2012–17)	National Centre for Disease Control	Sets up laboratory-based surveillance networks on AMR including laboratories of 30 state medical colleges in 24 states and Union Territories Monitoring antibiotic use and strengthening infection control	https://ncdc.gov.in/index1.php?lang=1&level=2&sublinkid=384&lid=344 [31]
Antimicrobial Resistance Surveillance and Research Network, 2013	Indian Council of Medical Research (ICMR)	Providing information on bacterial resistance incidences and patterns periodically from healthcare facilities	Walia et al., 2019 (https://www.ncbi.nlm.nih.gov/pmc/articles/PMC6563732/) [32]
Antimicrobial Stewardship Program (AMSP) Guideline, 2018	ICMR	Rational and optimum utilisation of antibiotics Standard guidelines of treatment and implementation of antibiotic policies Prescription auditing and streamlining Education and training of health care personnel	https://main.icmr.nic.in/sites/default/files/guidelines/AMSP_0.pdf [33]
Treatment Guideline for Antimicrobial Use in Common Syndromes, 2019	ICMR	Rational application of the antibiotics enlisted in the National List of Essential Medicines (NLEM) Guidelines on treating several infective diseases consistently	ICMR, 2019 (https://main.icmr.nic.in/sites/default/files/guidelines/Treatment_Guidelines_2019_Final.pdf) [34]
National Guidelines for Infection Prevention and Control in Healthcare Facilities, 2020	National Centre for Disease Control	Strengthening healthcare facilities (HCFs) in both private and public sectors to restrict the spread of AMR pathogens including patients, healthcare workers, and visitors	National Centre for Disease Control, Directorate General of Health Services, 2020 (https://www.mohfw.gov.in/pdf/National%20Guidelines%20for%20IPC%20in%20HCF%20-%20final%281%29.pdf) [35]

**Table 4:** Regulations and guidelines for the management of environmental pollutants to curb spread of AMR in the environment in India.

Rules and Regulations/Guidelines	Implementing Agency	Highlights	Sources
The Environment (Protection) Act, 1986		Environmental pollutants include any kind of harmful components in any state (solid, liquid, and gaseous), incorporating hazardous chemicals Laying down procedures and safeguards for the handling of hazardous substances	The Environment (Protection) Act, 1986 (https://www.indiacode.nic.in/bitstream/123456789/4316/1/ep_act_1986.pdf) [37]
Swachh Bharat Mission, 2017	Ministry of Drinking Water and Sanitation	Focused on the availability of safe drinking water, reduction in open defecation, and management of solid and liquid waste to reduce the incidence of diarrhoea, cholera, and other vector-borne diseases	Guideline for Swachh Bharat Mission, 2017 (https://swachhbharatmission.gov.in/sbmcms/writereaddata/images/pdf/Guidelines/Complete-set-guidelines.pdf) [38]
Biomedical Waste (BMW) Management Rules, 2016	Ministry of Environment, Forest and Climate Change (MoEFCC)	Rules for the treatment of waste including the generation, collection, storage, transportation, treatment, disposal, and handling of all forms of BMW from healthcare facilities, animal houses, veterinary institutions, blood banks, laboratories, Ayush, etc.	G.S.R 343(E) (https://dhr.gov.in/sites/default/files/Bio-medical_Waste_Management_Rules_2016.pdf) [39]
Antibiotic Residues in the treated effluent of Bulk Drug and Formulation Industry and CETP, 2020	Ministry of Environment, Forest and Climate Change (MoEFCC)	Sets the permissible residual limit of antibiotics from pharmaceutical manufacturing plants	G.S.R. 44(E) (http://moef.gov.in/wp-content/uploads/2020/01/finalization.pdf) [36]

## Data Availability

Data specific to this study are archived with the study team. It can be produced if necessary.

## Supplementary Materials

The following supporting information can be downloaded at: https://www.mdpi.com/article/10.3390/tropicalmed7110336/s1, Table S1: Search strategies of literature; Table S2: SWOT analysis findings from Plant agriculture; Veterinary; Biomedical waste & Pharmaceutical domains.

## References

[1] Kirchhelle C., Atkinson P., Broom A., Chuengsatiansup K., Ferreira J.P., Fortané N., Frost I., Gradmann C., Hinchliffe S., Hoffman S.J. (2020). Setting the standard: Multidisciplinary hallmarks for structural, equitable and tracked antibiotic policy. BMJ Glob. Health.

[2] Liguori K., Keenum I., Davis B.C., Calarco J., Milligan E., Harwood V.J., Pruden A. (2022). Antimicrobial Resistance Monitoring of Water Environments: A Framework for Standardized Methods and Quality Control. Environ Sci Technol. Environ. Sci. Technol..

[3] Klein E.Y., Van Boeckel T.P., Martinez E.M., Pant S., Gandra S., Levin S.A., Goossens H., Laxminarayan R. (2018). Global increase and geographic convergence in antibiotic consumption between 2000 and 2015. Proc. Natl. Acad. Sci. USA.

[4] Radhouani H., Silva N., Poeta P., Torres C., Correia S., Igrejas G. (2014). Potential impact of antimicrobial resistance in wildlife, environment and human health. Front. Microbiol..

[5] Sudha S., Mridula C., Silvester R., Hatha A.A. (2014). Prevalence and antibiotic resistance of pathogenic Vibrios in shellfishes from Cochin market. PLoS ONE.

[6] Tahrani L., Soufi L., Mehri I., Najjari A., Hassan A., Van Loco J., Reyns T., Cherif A., Mansour H.B. (2015). Isolation and characterization of antibiotic-resistant bacteria from pharmaceutical industrial wastewaters. Microb. Pathog..

[7] Kabir S.L., Asakura M., Shiramaru S., Pal A., Hinenoya A., Yamasaki S. (2015). Molecular identification and antimicrobial resistance profiles of Campylobacter strains of poultry origin in India with special emphasis on fluoroquinolone resistance. Asian J. Med. Health Res..

[8] Bardhan T., Chakraborty M., Bhattacharjee B. (2020). Prevalence of colistin-resistant, carbapenem-hydrolyzing proteobacteria in hospital water bodies and out-falls of West Bengal, India. Int. J. Environ. Res. Public Health.

[9] Taylor P., Reeder R. (2020). Antibiotic use on crops in low and middle-income countries based on recommendations made by agricultural advisors. CABI Agric. Biosci..

[10] Landers T.F., Cohen B., Wittum T.E., Larson E.L. (2012). A review of antibiotic use in food animals: Perspective, policy, and potential. Public Health Rep..

[11] Mutua F., Sharma G., Grace D., Bandyopadhyay S., Shome B., Lindahl J. (2020). A review of animal health and drug use practices in India, and their possible link to antimicrobial resistance. Antimicrob. Resist. Infect. Control.

[12] Patel I., Hussain R., Khan A., Ahmad A., Khan M.U., Hassalai M.A. (2017). Antimicrobial resistance in India. J. Pharm. Policy Pract..

[13] Bürgmann H., Frigon D., Gaze W.H., Manaia C.M., Pruden A., Singer A.C., Smets B.F., Zhang T. (2018). Water and sanitation: An essential battlefront in the war on antimicrobial resistance. FEMS Microbiol. Ecol..

[14] Aarti C., Khusro A., Arasu M.V., Agastian P., Al-Dhabi N.A. (2016). Biological potency and characterization of antibacterial substances produced by Lactobacillus pentosus isolated from Hentak, a fermented fish product of North-East India. Springerplus.

[15] Hanna N., Purohit M., Diwan V., Chandran S.P., Riggi E., Parashar V., Tamhankar A.J., Lundborg C.S. (2020). Monitoring of water quality, antibiotic residues, and antibiotic-resistant escherichia coli in the kshipra river in india over a 3-year period. Int. J. Environ. Res. Public Health..

[16] Bishwajit G., Ide S., Ghosh S. (2014). Social determinants of infectious diseases in South Asia. Int. Sch. Res. Notices.

[17] World Health Organization (2019). Monitoring and Evaluation of the Global Action Plan on Antimicrobial Resistance: Framework and Recommended Indicators. https://apps.who.int/iris/bitstream/handle/10665/325006/9789241515665eng.pdf?sequence=1&isAllowed=y.

[18] 2019 WHO AWaRe Classifcation Database of Antibiotics for Evaluation and Monitoring of Use. https://www.who.int/publications/i/item/WHOEMPIAU2019.11.

[19] The Insecticide Act 1968. 46 (Ind.), Adopted on 2 September 1968. https://legislative.gov.in/sites/default/files/A1968-46.pdf.

[20] Central Insecticide Board & Registration Committee (2021). Major Uses of Pesticides. Ministry of Agriculture & Farmers Welfare: Government of India. http://ppqs.gov.in/sites/default/files/2._mup_fungicide_upto_30.06.2021.pdf.

[21] Center for Disease Dynamics, Economics & Policy Antibiotic Use and Resistance in Food Animals Current Policy and Recommendations. https://cddep.org/wp-content/uploads/2017/06/india_abx_report-2.pdf.

[22] Kumar H.C., Hiremath J., Yogisharadhya R., Balamurugan V., Jacob S.S., Reddy G.M., Suresh K.P., Shome R., Nagalingam M., Sridevi R. (2021). Animal disease surveillance: Its importance & present status in India. Indian J. Med. Res..

[23] Coastal Aquaculture Authority (2005). Guidelines for regulating Coastal Aquaculture (Annexure-I), CAA Rules. http://caa.gov.in/uploaded/doc/Guidelines-Englishnew.pdf.

[24] Export Inspection Council (2011). Milk Product: Residue Monitoring Plan (RMP) for Export to EU, Year 2011-12.

[25] Export Inspection Council (2019). Honey: Residue Monitoring Plan (RMP), Year 2019-20.

[26] Marine Products Export Development Authority (MPEDA) National Residue Control Plan (NRCP). https://mpeda.gov.in/?page_id=1088.

[27] Srivastava R.K. (2011). National Policy for Containment of Antimicrobial Resistance India.

[28] Central Pollution Control Board (2021). Guidelines for Environmental Management of Dairy Farms and Gaushalas.

[29] Central Pollution Control Board (2021). Environmental Guidelines for Poultry Farms.

[30] Vijayan K.K. (2016). CIBA’S Initiatives in promoting the responsible use of drugs and chemicals in Indian aquaculture. Responsible Use of Antimicrobials in Indian Aquaculture: Opportunities and Challenges.

[31] National Programme on AMR Containment (2012-17). https://ncdc.gov.in/index1.php?lang=1&level=2&sublinkid=384&lid=344.

[32] Walia K., Madhumathi J., Veeraraghavan B., Chakrabarti A., Kapil A., Ray P., Singh H., Sistla S., Ohri V.C. (2019). Establishing antimicrobial resistance surveillance & research network in India: Journey so far. Indian J. Med. Res..

[33] Antimicrobial Stewardship Program (AMSP) Guideline. https://main.icmr.nic.in/sites/default/files/guidelines/AMSP_0.pdf.

[34] Indian Council of Medical Research (ICMR) (2019). Treatment Guidelines for Antimicrobial Use in Common Syndromes.

[35] National Centre for Disease Control, Directorate General of Health Services (2020). National Guidelines for Infection Prevention and Control in Healthcare Facilities.

[36] Gazette of India (2020). Gazette Notification G.S.R. 44(E).

[37] The Environmental (Protection) Act, 1986. 29 (Ind.). https://www.indiacode.nic.in/bitstream/123456789/4316/1/ep_act_1986.pdf.

[38] Ministry of Drinking Water and Sanitation (2017). Guidelines for Swachh Bharat Mission (Gramin). https://swachhbharatmission.gov.in/sbmcms/writereaddata/images/pdf/Guidelines/Complete-set-guidelines.pdf.

[39] Gazette of India (2016). Gazette Notification G.S.R. 343(E). Gazette of India: New Delhi. https://dhr.gov.in/sites/default/files/Bio-medical_Waste_Management_Rules_2016.pdf.

[40] Centre for Science and Environment Antibiotic Misuse in Crops. https://www.cseindia.org/antibiotic-misuse-in-crops-9797.

[41] Department of Agriculture, Cooperation & Farmers’ Welfare Annual Report 2020–2021. https://agricoop.nic.in/sites/default/files/Web%20copy%20of%20AR%20%28Eng%29_7.pdf.

[42] Rai N., Limbu A.K., Joshi A. (2020). Impact of Trichoderma sp. in agriculture: A mini-review. J. Biol. Today’s World.

[43] Lally R.D., Galbally P., Moreira A.S., Spink J., Ryan D., Germaine K.J., Dowling D.N. (2017). Application of endophytic Pseudomonas fluorescens and a bacterial consortium to Brassica napus can increase plant height and biomass under greenhouse and field conditions. Front. Plant Sci..

[44] (2017). Biosecurity Manual for Sugarcane Producers—A Guide to Farm Biosecurity Measures to Reduce the Risks of Weeds, Pests and Diseases Impacting Production (Version 1.0).

[45] Gandra S., Joshi J., Trett A., Lamkang A.S., Laxminarayan R. (2017). Scoping Report on Antimicrobial Resistance in India.

