# Supplementary material for: Existing Policies/Guidelines on the Environmental Dimension of Antimicrobial Resistance in India: An Insight into the Key Facets through Review and SWOT Analysis
Source: Trop Med Infect Dis. 2022 Oct 29;7(11):336. doi: 10.3390/tropicalmed7110336 (PMC9699572; doi:10.3390/tropicalmed7110336)
Supplement: Supplementary file 1 [file tropicalmed-07-00336-s001.zip › tropicalmed-1933099-supplementary.pdf]

Tropical Medicine and Infectious Disease

MDPI

Supplementary Materials: Existing Policies/Guidelines on the Environmental Dimension of Antimicrobial Resistance in India: An Insight into the Key Facets through Review and SWOT Analysis

Table S1: Search strategies of literature (Supplement)

Search domain	Search strategy
Plant Agriculture	((((((((((antimicrobials) OR antibiotics) AND policies) AND guidelines) AND legislation) and crop cultivation) OR crop production) AND environment) AND India [Title]
Animal health	((((((((((antimicrobials) OR antibiotics) AND policies) AND guidelines) AND legislation) AND livestock rearing) OR poultry farming) OR cattle farming) OR dairy farming) OR aquaculture) OR swine farming) OR food animals) AND environment) AND India [Title]
Human Health	(((((((antimicrobials) OR antibiotics) AND policies) AND guidelines) AND legislation) AND human health) OR public health) AND environment) AND India [Title]
Environment	(((((((antimicrobials) OR antibiotics) AND policies) AND guidelines) AND legislation) AND biomedical waste) OR hospital waste) OR laboratory waste) OR hospital effluents) OR pharmaceutical waste) AND environment) AND India [Title]

Table S2. SWOT analysis findings from Plant agriculture; Veterinary; Biomedical waste & Pharmaceutical domains.

Domain	Strengths	Weaknesses	Opportunities	Threats
Plant agriculture	Presence of an act related to prohibition of the importing, manufacturing, and selling of insecticides incorporation of some antibiotics such as streptomycin and tetracycline; Guidelines for prescribed formulation of fungi-cides and their combined use, including anti-biotic formulation, for specific plants	Lack of resources within the system for rendering services to more farmers; Lack of knowledge among farmers; Desire to make profit; Lack of structure for capacity building of farmers	Scope of introduction of structured capacity building programs for farmers; Scope of inclusion of policies on financial protection of farmers incase of crop failure due to infection	Availability of antimicrobial formulations for use in plant agriculture in market; Absence of surveillance on sales of such preparation at market may currently act as a threat for optimizing use of antimicrobials in plant/agriculture
Veterinary	Presence of acts, regulations, guidelines and surveillance;	Lacking of provisions for quality check of domestic products such as even seeds; Absence of structured	Presence of strict surveillance on use of antimicrobials for exportable food animals is the opportunity to introduce similar	Absence of any provision for controlling the high cost of available growth promoters of animals acts as a threat; Availability of

		capacity building program provisions for farmers; absence of surveillance mechanisms for regulation of administration of antibiotics, mechanism for regular checking of antibiotic residues in domestic food animals ;Lack of monitoring of phasing out critically important antibiotics for humans as growth promoters are identified to be major weaknesses; lack of coordination among different authorized bodies like the Bureau of Indian Stand-ards (BIS) and the Food Standards and Safety Authority of India (FSSAI) regarding the list of antibiotics to be used within a limit	mechanisms for quality check in domestic food animals	antimicrobials in the market
Biomedical waste & Pharmaceuticals	Presence of few policies in both the sectors	Policies are mostly related to disposal and management of hazardous substances and other solid and liquid wastes; Lack of clarity on process of disposal and management of antimicrobial drugs and related waste generated from hospitals, community, manufacturing units;	Ministry of Environment, Forest and Climate Change (MoEFCC) has published the permissible level of antibiotic residues in effluent very recently and this may work as opportunity to control spread of AMR through discharge of effluents from pharmaceutical companies	Over the counter sale of antimicrobials; 'However, lack of capacity among professionals across sectors to recognize containment of AMR as multi sector initiative is posing a major threat in current scenario
